# Updated Treatment for Calcium Pyrophosphate Deposition Disease: An Insight

**DOI:** 10.7759/cureus.3840

**Published:** 2019-01-07

**Authors:** Shumaila M Iqbal, Sana Qadir, Hafiz M Aslam, Madiha A Qadir

**Affiliations:** 1 Internal Medicine, University at Buffalo / Sisters of Charity Hospital, Buffalo, USA; 2 Internal Medicine, S & A Pediatrics, Parsippany, USA; 3 Internal Medicine, Seton Hall University / Hackensack Meridian School of Medicine, Trenton, USA; 4 Internal Medicine, Jinnah Sindh Medical University, Karachi, PAK

**Keywords:** calcium pyrophosphate deposition disease, calcium pyrophosphate crystal, arthritis, colchicine, immunomodulants, nsaid

## Abstract

Calcium pyrophosphate disease (CPPD) is caused by the deposition of calcium pyrophosphate (CPP) crystals in the joint tissues, particularly fibrocartilage and hyaline cartilage. CPP crystals trigger inflammation, causing local articular tissue damage. Our review article below covers different aspects of CPPD. It discusses how CPPD can manifest as different kinds of arthritis, which may be symptomatic or asymptomatic. The metabolic and endocrine disease associations and routine investigations used in the diagnostic workup are briefly reviewed. Conventional and newer therapies for the treatment of CPPD are outlined. Overall, this extensive review would provide an updated insight to clinicians for evidence-based treatment of CPPD.

## Introduction and background

Calcium pyrophosphate deposition disease (CPPD) is caused by the deposition of calcium pyrophosphate (CCP) crystals in the articular cartilage, resulting in inflammation and degenerative changes in the affected joint. CPPD is a collective term that comprises all the various forms of CPP crystal-induced arthropathies. The acute arthritis attack caused by CPP crystals deposition triggers the same inflammatory reaction in the joint tissues as do the monosodium urate crystals in gout patients. The symptoms include pain, tenderness, stiffness, redness, warmth and decreased range of motion in the affected joint.

Gender and race does not seem to have an impact on developing non-urate gout, it affects men and women equally. The risk factors of CPPD are prior joint trauma or surgery [1], old age, history of gout, hypothyroidism, hyperparathyroidism, familial tendency, hemochromatosis, hemophilia and metabolic derangements comprising hypophosphatemia, hypomagnesemia or hypercalcemia [2].

In comparison to true gout, limited evidence-based researches have been done on CPP-related arthropathies, particularly there is a lack of randomized controlled trials. To date, no medicine has been discovered yet that targets the interruption of rising concentration of CPP crystals specifically in the joints, and therefore, principally symptomatic treatment is given. The aim of our article was to enlighten the different treatment strategies used to manage the various types of calcium crystal arthropathies.

## Review

Pathogenesis of CPPD

The hydrolysis of adenosine triphosphate (ATP) generates energy and yields a compound called inorganic pyrophosphate (PPi) [3]. This PPi accumulates in the extracellular matrix of cartilage. The degradation of PPi is catalyzed by inorganic pyrophosphatase in the fibroblasts of chondrocytes. It is important to maintain equilibrium between the production and degradation of PPi to maintain normal homeostasis of PPi [3]. Either enhanced production or decreased removal of PPi in cartilage favors the excess PPi to bind with calcium, leading to the precipitation of CPP crystals in joint tissue [3].

The modern era of advanced molecular genetics has identified the familial forms of CPPD with an autosomal dominant (AD) pattern of inheritance most of the time, with a relatively younger age of onset [4]. Mutations in the gene ANKH on chromosome 5p have a significant impact on familial CPPD [5]. ANKH gene codes for a transmembrane transport protein called ANK protein, which regulates the transportation of extracellular and intracellular PPi to maintain normal cartilage homeostasis [6]. ANKH mutation results in increased transcription and the translation of ANK transport protein implying gain of function. The consequence is increased efflux of PPi into the extracellular space of chondrocyte promoting CPP crystal formation [6].

The major structural proteins of cartilage are encoded in a gene named COL2A1 located on chromosome 12q; its mutation can result in defective collagen production, causing another severe form of familial chondrocalcinosis [6]. According to a genetic study, some cases of familial CPPD have also been genetically linked to chromosome 8q [4]. Chondrocalcinosis in those patients was secondary to cartilage matrix degeneration by severe osteoarthritis (OA).

Immune system attempts to clear up the CPP crystal deposits in the chondrocytes by performing phagocytosis with the help of local monocytes and macrophages. CPP crystals specifically elicit the formation of NOD-like receptor, pyrin domain containing 3 (NLRP3) inflammasome [7], which promotes caspase-1 activation and the initiation of inflammatory cascade releasing pro-inflammatory cytokines, e.g., interleukin-1-beta and interleukin-18. Another chemokine named interleukin-8 is also produced by the activated macrophages, which induces the migration of neutrophils into the articular tissues further up-regulating the inflammatory reaction. Understanding the molecular level of CPP-induced tissue inflammation is essential to recognize the potential of therapeutic interventions targeting inhibition of inflammation and to prompt exploration of more effective drugs.

Clinical presentations of CPPD

CPPD-related arthropathies are caused by the precipitation of CPP crystals in the connective tissues of joints such as fibrocartilage or hyaline cartilage and synovial membrane. They can be asymptomatic or manifest in the form of different clinical syndromes. The European League Against Rheumatism (EULAR) has coined the term “CPPD” to include all the various phenotypes of calcium pyrophosphate occurrences (Table 1) [8].

**Table 1:** Clinical Presentations of CPPD CPPD: calcium pyrophosphate deposition disease, CPP: calcium pyrophosphate, PIP: proximal interphalangeal joint, MCP: metacarpophalangeal joint

Clinical Presentations	
Asymptomatic chondrocalcinosis	Pathological calcification of joint cartilage. Usually remains asymptomatic.
Acute CPP crystal arthritis	CPP crystals deposition in articular cartilage and synovial membrane eliciting an acute inflammatory process. Distribution is usually mono-articular. The most commonly affected joint is knees.
Chronic CPP crystal inflammatory arthritis	Tissue deformities caused by chronic deposition of CPP crystals.
Pseudo-osteoarthritis	Characterized by the concomitant presence of pseudogout with osteoarthritis.
Pseudo-rheumatoid arthritis	Poly-articular and symmetrical in distribution especially affecting PIP and MCP joints. Morning stiffness with an elevated level of inflammatory markers. Low titers of RF have been isolated in 10% cases.
Pseudo-neuropathic arthropathy	Radiologically resembles Charcot joint without any underlying neurological conditions and normal results for nerve conduction study test.

The clinical presentations of CPPD have been elaborately described below:

*Asymptomatic Chondrocalcinosis* (Lanthanic)

Chondrocalcinosis refers to the pathological calcification in cartilage, often seen in elderly age group (>80 years) and patients with a history of joint trauma. Asymptomatic chondrocalcinosis is usually detected as an incidental X-ray finding in a symptom-free patient. Generally, it has no clinical significance; however, according to a survey, patients with radiographic chondrocalcinosis are more likely to give joint complaints upon taking complete history as compared to a control group of similar age without chondrocalcinosis [9].

Chronic CPP Crystal Inflammatory Arthritis

Chronic CPP crystal inflammatory arthritis is a mild version of acute arthritis. It presents with dull pain, mild swelling, morning stiffness and a decreased range of motion. The presentation of chronic CPP crystal arthritis is mostly bilateral, symmetrical, involving multiple joints [10]. The episode of chronic arthritis might last for many months, causing significant deformation of the articular tissues. Due to some overlapping symptoms with rheumatoid arthritis, it is often misdiagnosed as rheumatoid arthritis; therefore, it is important to perform synovial fluid analysis to reach an accurate diagnosis [11].

Pseudo-osteoarthritis

Two varying hypotheses have been proposed on the etiology of pseudo-OA; it is unclear whether primary OA predisposes to the development of CPPD or CPP crystals initiate the event by causing joint damage. In advanced stages of OA, the deposition of CPP crystals is observed in damaged joint cartilage. It involves usually a knee joint, metacarpophalengeal (MCP) joints, wrist and shoulder joint. Recent studies have demonstrated the presence of another type of crystals in OA cartilage called basic calcium phosphate (BCP), which is uniquely related to OA; therefore their presence can confirm this particular form of CPPD [12-13]. It has been found that the concentration of BCP crystals strongly correlates with the severity of OA; thus, agents targeting BCP crystals can give promising results [12-13]. 

Pseudo-rheumatoid Arthritis

This presentation of CPPD is relatively less common. It is often misdiagnosed as true rheumatic arthritis (RA) due to many overlapping classical features [14]. Patients of pseudo-RA typically present with polyarticular joint pain and swelling (especially proximal interphalangeal joint and MCP joint), showing symmetrical distribution, accompanied with morning stiffness and raised erythrocyte sedimentation rate (ESR). Low titers of rheumatic factor (RF) can also be isolated in 10% cases, further adding to diagnostic confusion. In contrast to pseudo-RA, classic RA can be differentiated by the presence of high titers of RF and a more specific antibody called anti-cyclic citrullinated peptide (CCP) antibodies.

Pseudo-neuropathic Arthropathy

Pseudo-neuropathic arthropathy is an unusual subtype of CPPD-related arthropathy, which is not fully understood yet. The clinical and radiological picture of pseudo-neuropathic arthropathy resembles neuropathic Charcot’s joint, despite absent underlying neurological conditions such as diabetic peripheral neuropathy, syringomyelia and tabes dorsalis [15]. It causes pronounced joint damage in a relatively short period of time. Regardless of having normal nerve conduction studies and electromyography, patients present with severe painful monoarthritis, commonly involving the knee joint.

Acute CPP Crystal Arthritis (Pseudogout)

Acute CPP crystal arthritis is the most widely encountered manifestation of CPPD. The accumulation of CPP crystals in the articular cartilage and synovial membrane trigger the immune system to elicit an inflammatory reaction, resulting in arthritis and synovitis, respectively. It is characterized by a sudden onset of pain, swelling and tenderness in the affected joint. The attacks of acute arthritis are self-limiting and usually last days to weeks [11]. Patients may stay asymptomatic between these episodes. Surgeries or serious medical illness such as stroke, myocardial infarction can trigger acute flares of arthritis [11]. Pseudogout arthritis may begin as a monoarthritis in the initial course of the disease, but it usually progresses to a polyarticular form in about 2/3rd patients. Acute CPP crystal arthritis follows an asymmetrical pattern in most of the cases. It frequently affects large joints, most commonly the knees. Other less frequently involved joints are the wrist, ankle, first MTP joint and shoulder joint.

Diagnosis

**Figure 1:**
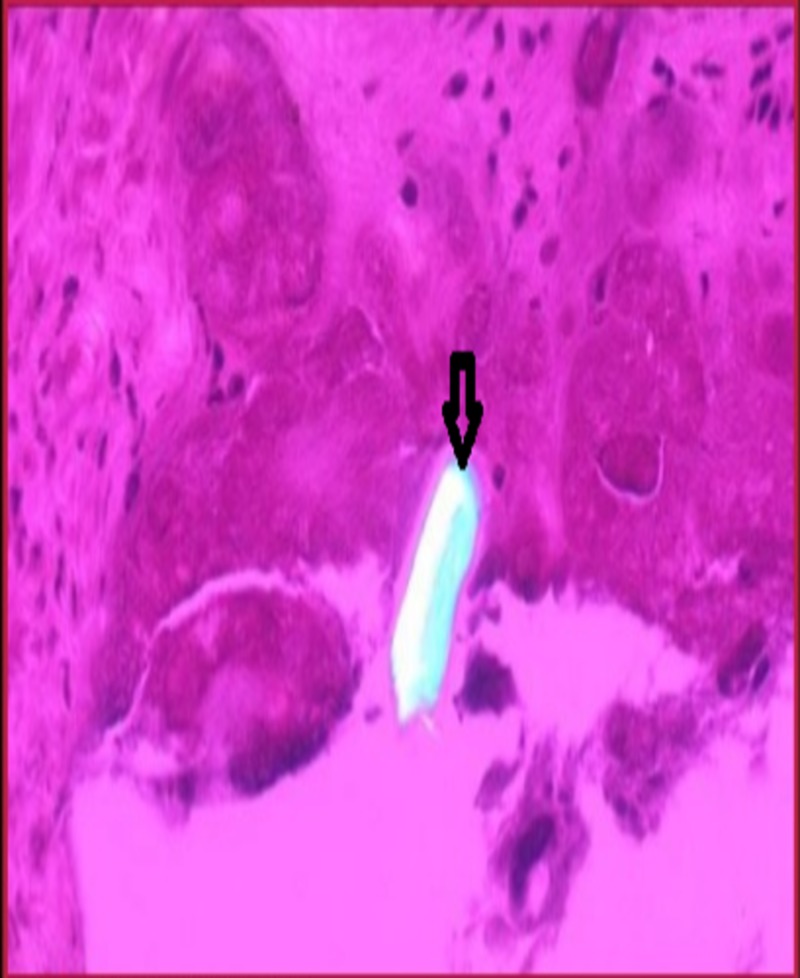
Showing weakly positive birefringent crystals of calcium pyrophosphate under polarized light.

Clinically it is difficult to distinguish CPPD arthropathies from true gout; therefore, the definitive diagnosis is confirmed by performing arthrocentesis and synovial fluid analysis [16]. In this technique, the synovial fluid aspired from the affected joint is examined under a polarizing light microscope. The characteristic features of CPP crystals are weak positive birefringent crystals, mostly rhomboid- or rod-shaped (Figure 1), appearing blue in color when stained with H&E stain. Upon visualizing these characteristics under the polarizing microscope, CPPD-related arthropathies are diagnosed.

**Figure 2:**
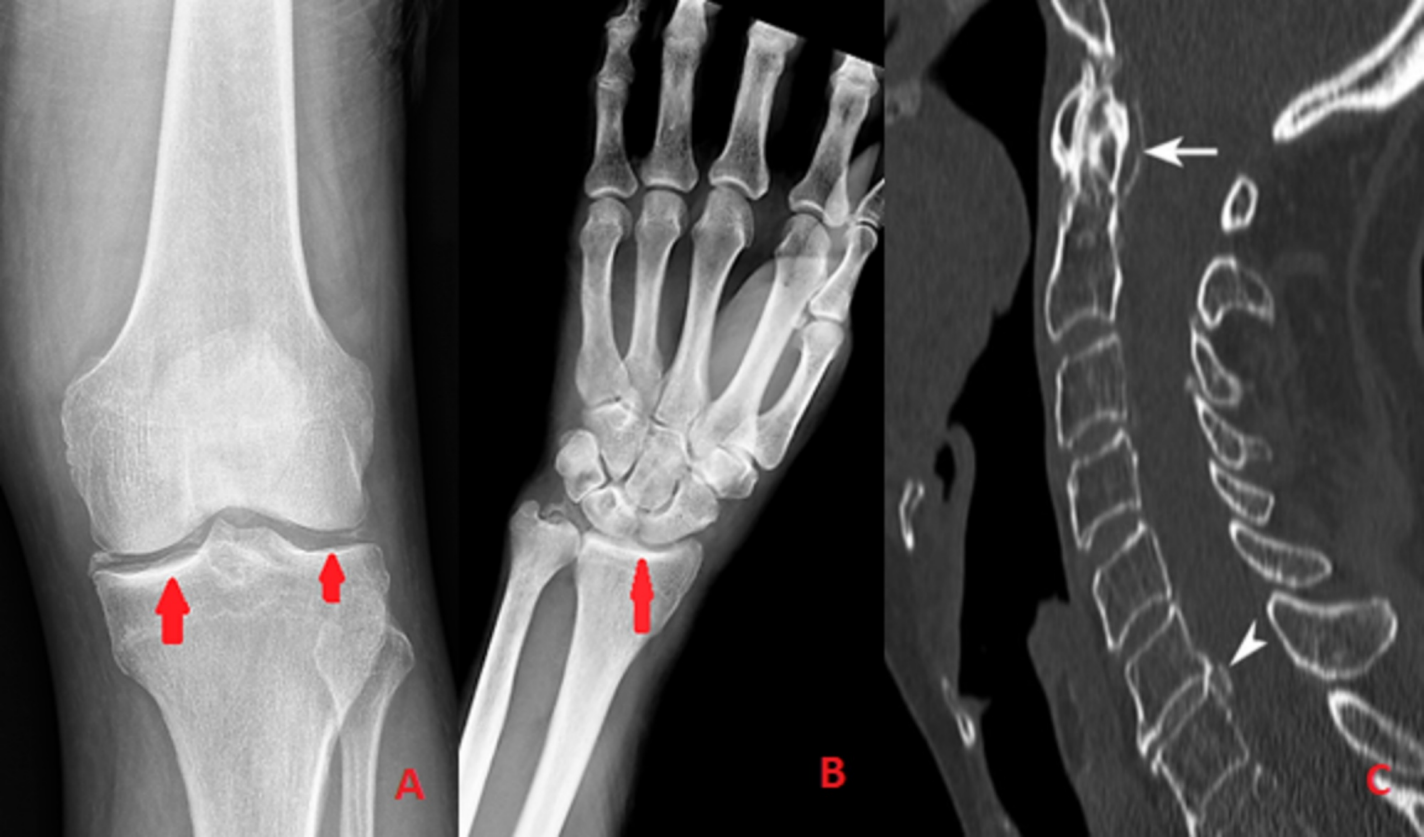
A & B showing chondrocalcinosis evident on Xray, C showing crowned dense syndrome.

Another important diagnostic tool for this CPPD is plain X-ray. X-ray can detect calcifications in the cartilage (chondrocalcinosis) and also reveal the extent of damage in the joint (Figure 2). Other radiographic features that can be visualized are osteophytes, the narrowing of joint space and subchondral cysts [2]. Additional radiographic modalities, such as ultrasound, MRI and CT can be useful in patients with atypical sites of CPP crystal deposition, e.g., atlanto-occipital joint and spine (Crowned dens syndrome; Figure 2) [11]. Among them, ultrasound is the most sensitive diagnostic tool in detecting tissue inflammation and deposits of CPP, even without evident calcification on plain X-ray. Usually, the diagnosis is established on the basis of both radiographic and synovial fluid analysis.

The assessment of the underlying metabolic pathologies causing CPPD, such as hyperparathyroidism, hypothyroidism and hemochromatosis, is equally important. They can be screened by measuring the serum levels of thyroid hormone, parathyroid hormone and alkaline phosphatase, in addition to measuring the serum calcium, phosphate, iron and magnesium levels.

Interventions and treatment options

The primary goals for treating CPPD is to control inflammation and prevent acute flares. Treatment is not indicated for patients with asymptomatic (lanthanic) chondrocalcinosis. CPPD patients with associated arthritis can use general measures to reduce joint stiffness and maintain mobility; such as exercise, weight reduction and wearing joint support aids. Pharmacological management for CPPD is summarized below in Table 2 and has been mentioned extensively under the subsequent section.

**Table 2:** Updated treatment for calcium pyrophosphate deposition disease CPPD: calcium pyrophosphate deposition disease, CPP: calcium pyrophosphate, COL: colchicine, HCQ: hydroxychloroquine, MTX: methotrexate, NSAID: nonsteroidal anti-inflammatory drug, PC: phosphocitrate, PolyP: polyphosphate

Updated Treatment for Calcium Pyrophosphate Deposition Disease:	
Conventional Therapies:	
NSAID	Low dose Naproxen and Indomethacin. Effective for treating flares of CPPD and reduce the frequency of recurrent episodes of flare.
Steroid (oral, intra-articular, intra-muscular)	Effective only in flares. Oral steroids should be preferred when joint involvement is polyarticular; intra-muscular steroids when contraindication to use oral steroids and joint involvement is polyarticular; intra-articular steroids when joint involvement is mono- or oligoarticular.
COL (oral, intravenous)	Effective in flares when used in combination with NSAIDs. It is also beneficial as prophylaxis for preventing recurrent flares. Oral COL should be preferred over the intravenous form. If contraindications present to use oral COL, then intravenous COL solution should be pre-diluted with 0.9% NaCl prior to infusion.
Other Treatment Considerations:	
MTX	Has an anti-inflammatory and immunosuppressive property, and thus effective in flares of CPPD when it is resistant to treatment with conventional therapies and or contraindications for conventional therapies are present. It also prevents recurrent flares.
HCQ	Effective in chronic CPPD-related arthropathies
Interleukin-1 receptor antagonists (Anakinra)	Effective for CPPD flares and preventing its recurrent episodes. Can be utilized when contraindications are present for conventional therapies and or if acute disease process is resistant to treatment with classic treatment approach.
Radiosynovectomy	Newer radioactive technique to remove inflamed synovium. Patients with CPPD secondary to hemophilia are best candidates for it.
Potential Future Treatments for CPPD	
Anti-crystal agents (probenecid, PC and PolyP)	Effective by preventing organization of CPP crystals

Conventional drugs for treatment and prevention of acute flares related to CPPD

Based on the clinical trials, it is recommended to treat the attacks of acute CPPD arthritis in the same way as true gout is treated. In certain cases with significant swelling and pain, joint aspiration is performed to reduce the pressure within the joint; it has both diagnostic and therapeutic value [16-17]. Some nonpharmacological measures such as applying ice packs and taking rest can also temporarily relieve pain and swelling. Anti-inflammatory drugs such as NSAIDs and glucocorticoids remain the mainstay of treatment. They can terminate on occasion acute attacks and relieve pain but cannot modify the course of the disease. Colchicine remains the conventional treatment for preventing recurrent episodes of acute flares.

Nonsteroidal Anti-inflammatory Drugs

NSAIDs, notably ibuprofen and indomethacin, are given in low doses to suppress inflammation. If an acute flare is untreated, the symptoms can last for a longer time period. NSAIDs act by blocking an enzyme called cyclooxygenase (COX), which has a central role in the production of inflammation-causing compounds known as prostaglandins. Low-dose NSAIDs not only abort the current attack but also help reduce the frequency of further episodes of acute flares. However, despite the good efficacy of NSAIDs, they are also well known for many potential side effects and drug interactions, and therefore, it is recommended to discontinue them as soon as the pain subsides. The long-term use of NSAIDs can result in kidney injury and peptic ulcers, which can be countered by monitoring renal function (blood creatinine) and prescribing proton pump inhibitors (PPIs), respectively. NSAIDs should be avoided in patients with an increased risk of upper GI bleeding and renal or hepatic failure.

Corticosteroids

Corticosteroids (CS) are the drug of choice in patients with contraindication to NSAIDs and colchicine [11]. CS are very effective and rapid-acting; their response can be noticed within 24 hours of beginning therapy. It is administered orally, intra-articularly or intra-muscularly. An intra-articular route is more preferred because of its localized action and better tolerance in elderly patients with multiple comorbidities, but an intra-articular CS is useful only when arthritis is oligoarticular, involving one or two joints. Oral steroids are given as prednisone or methylprednisolone; they work best for patients with severe polyarticular attacks. Usually, a short course of tapering oral steroids is recommended, because it is associated with many systemic side effects such as weight gain, frequent infections due to immunosuppression, acne, muscle weakness, anxiety and osteoporosis on long-term use. Oral CS can also exacerbate pre-existing diabetes by promoting hyperglycemia. In a prospective study performed on 14 patients who presented with an acute flare of pseudogout, intra-muscular triamcinolone injection provided an effective treatment to the patients. Thus, an intra-muscular steroid injection can be considered a reasonable alternative therapy in patients with an acute flare of CPPD when NSAIDs are contraindicated and joint involvement is polyarticular making utilization of intra-articular CSs impractical [18].

Colchicine

Colchicine (CL) has been proven to be a miracle in preventing acute flares [19]. It works by interfering with the polymerization of microtubules, necessary for the migration of neutrophils to the inflammation site, in addition to the inhibition of assembling the inflammasome complex [20]. COL is most preferably given through an oral route because it is highly caustic when administered intravenously and its extravasation can cause irritation and tissue necrosis. Nevertheless, in case of severe gastritis where NSAIDs and oral colchicine should be avoided, the use of intravenous colchicine pre-diluted with 0.9% NaCl has been reported to be equally efficacious [21].

Other treatment considerations

Certain patients fail to respond to the above-mentioned conventional drugs. Hence, some disease-modifying antirheumatic drugs (DMARDs) such as methotrexate and hydroxychloroquine and other medicines have been considered to treat refractory cases of CPPD arthritis on the basis of randomized controlled trials conducted on small scale.

Methotrexate

Although, the mechanism of action of methotrexate (MTX) with reference to treating CPPD is poorly understood; it is believed that it is because of its anti-inflammatory and immunosuppressant properties. For its immunosuppressive property, MTX is particularly fruitful for pseudo-rheumatic presentation and polyarticular arthropathies with recurrent acute attacks [11]. Its anti-inflammatory role was evident in a study performed on five patients. Their acute CPPD was resistant to classic treatment with NSAIDs and steroids. Low-dose MTX (5-20 mg/week) significantly reduced the amount of pain, swelling of joints and the serum levels of inflammatory biomarkers [22]. Another observational study performed on 10 patients showed MTX being beneficial for treating acute inflammation in patients with treatment-resistant CPPD by conventional therapies [23].

Hydroxychloroquine

Hydroxychloroquine (HCQ) is originally an anti-malarial drug that can be used as an adjuvant drug [24]. Several mechanisms of action have been suggested for HCQ in context to the treatment of CPPD, all of which signify its capability to immunomodulate and reduce inflammation. HCQ blocks the activity of T-cells, reduces the release of various cytokines (interleukin-1, interleukin-6 and tumor necrosis factor-alfa). It has also demonstrated to inhibit the activity of matrix metalloprotease in experimental animals. In a double-blinded, prospective six-month trial, HCQ was found to be beneficial specifically for chronic CPPD-related arthropathies [25].

Interleukin-1 Receptor Antagonists

Interleukin-1 receptor antagonists, namely anakinra, are recombinant genetically modified biopharmaceutical drugs that competitively bind to the interleukin-1 receptor, preventing the action of interleukin-1, a very prominent cytokine in the pathway of inflammation; thus halting the assembly of an inflammasome complex. In a report of 16 cases where the use of NSAID was contraindicated and disease was refractory to treatment with steroids, the utilization of anakinra showed beneficial response [26]. An another reported case mentioned anakinra to be beneficial for both prophylaxis and treatment of CPPD in patients with renal failure where the use of NSAIDs was contraindicated [27]

Two other recombinant drugs that belong to this class are canakinumab, a neutralizing monoclonal antibody directed against the interleukin-1 receptor, and rilonacept, also known as interleukin-1 trap, a soluble decoy receptor fusion protein. They can be used as an effective alternative to CS-resistant cases [11]. Another immunomodulating agent tumor necrosis factor-alfa inhibitor has not been successful to benefit CPPD patients.

Magnesium

A double-blind, placebo-controlled randomized clinical trial (RCT) conducted on magnesium carbonate in chronic CPPD arthropathy patients proved to bring improvement in joint pain and stiffness [28]. In vitro studies have shown that CPP crystals can be solubilized by magnesium (Mg) along with the inhibition of their crystal growth. Besides this fact, Mg can also be used as a supplement in patients with CPPD secondary to Mg deficiency. 

Hyaluronan

Sodium hyaluronan is a viscosupplementation approved to treat OA. It is a naturally occurring synovial fluid component that allows the gliding of bones upon each other smoothly. Based on this chondroprotective feature of hyaluronan, it is injected intra-articulary to increase joint mobility and improve joint function when conventional drugs fail [11,29]. Generally, it is well tolerated in patients without any adverse effects; but some patients have been reported to develop acute pseudo-septic arthritis hours after shots of hyaluronic acid [29].

Radiosynovectomy

Radioactive synovectomy is a minimally invasive technique that involves the injection of small radioactive particles intra-articularly to remove inflamed synovium. Although it can be used to treat different kinds of arthritis [30], patients with CPPD secondary to hemophilia are the best candidates for radiosynovectomy, especially with repeated joint bleeding [31]. Radiosynovectomy is a safe, cost-effective and efficient therapeutic option with good response rate and low radiation exposure.

Surgery

In particular, pseudo-neuropathic arthropathy may be benefited by the surgical replacement of damaged joints by a prosthesis (arthroplasty) to correct the deformity.

Treatment of comorbidities

Disease-specific treatment should be provided in patients with underlying secondary metabolic diseases such as hyperparathyroidism, hypothyroidism, hypophosphatemia and hypomagnesemia, despite the fact that it will not bring a prominent improvement in the arthropathy [11]. A retrospective study confirms that even parathyroidectomy had no impact on preventing future attacks or decreasing preexisting cartilage calcification [11].

Potential future treatments for CPPD-associated pseudogout

Theoretically, any pharmacological or surgical approach that works to inhibit the origination of CPP crystals or promotes the dissolution of crystals can cure CPPD arthropathies; hence, more research is required in this area to explore better interventions. The manual removal of chondrocyte calcification by surgery still remains an experimental procedure.

Anti-crystal agents

The availability of high levels of free inorganic phosphate in the extracellular matrix of chondrocytes seem to lay the foundation of calcium crystals, therefore using pharmacological agents that can lower the free phosphate levels such as probenecid, phosphocitrate (PC) and polyphosphate (polyP) can prevent CPP crystal formation [11]. The mechanism by which probenecid counters CPP crystal development is thought to be due to its inhibitory action on TGF beta-1, an important stimulant of NTPPPH enzyme required for pyrophosphate synthesis. Besides, a formulation of PC has been demonstrated to be a potent anti-mineralization agent on an animal model, and thus it can help reduce calcium deposits [32-33]; but no data are available on humans. Another agent that can be used to dissolve the CPP crystals is polyP; they have the potential to inhibit mineralization locally. However, at present, the action of these agents is a theoretical possibility, which needs to be confirmed. The severity of OA-related CPPD (pseudo-OA) is thought to be related to the concentration of BCP crystals, and it is hoped that discovering drugs that therapeutically target BCP crystal precipitation can help treat pseudo-OA [13].

## Conclusions

CPPD is an umbrella term encompassing all the various clinical subsets of CPP crystal-related arthropathies. NSAIDs, CSs and COL still remain the standard drugs to treat acute pseudo-gout; but unfortunately, they have been observed to be less successful in treating chronic cases. To date, no specific treatment strategy has been discovered that can modify the disease or stop CCP crystal formation, and therefore, further research studies and clinical trials on other potential drugs should be done on a larger scale.

## References

[1] Fisseler-Eckhoff A, Mu¨ller K (1992). Arthroscopy and chondrocalcinosis. Arthroscopy.

[2] Kleiber Balderrama C, Rosenthal A, Lans D, Singh J, Bartels C (2017). Calcium pyrophosphate deposition disease and associated medical comorbidities: a national cross-sectional study of US veterans. Arthritis Care Res (Hoboken).

[3] Caswell A, Guilland-Cumming DF, Hearn PR, McGuire MK, Russell RG (1983). Pathogenesis of chondrocalcinosis and pseudogout. Metabolism of inorganic pyrophosphate and production of calcium pyrophosphate dihydrate crystals. Ann Rheum Dis.

[4] Baldwin CT, Farrer LA, Adair R, Dharmavaram R, Jimenez S, Anderson L (1995). Linkage of early-onset osteoarthritis and chondrocalcinosis to human chromosome 8q. Am J Hum Genet.

[5] Andrew L, Brancolini V, Serrano L (1999). Refinement of the chromosome 5p locus for familial calcium pyrophosphate dihydrate deposition disease. Am J Hum Genet.

[6] Zaka R, Williams C (2005). Genetics of chondrocalcinosis. Osteoarthritis and cartilage.

[7] Franchi L, Warner N, Viani K, Nuñez G (2009). Function of Nod-like receptors in microbial recognition and host defense. Immunol Rev.

[8] Zhang W, Doherty M, Bardin T (2011). European League Against Rheumatism recommendations for calcium pyrophosphate deposition. Part I: terminology and diagnosis. Ann Rheum Dis.

[9] Ellman M, Levin B (1975). Chondrocalcinosis in elderly persons. Arthritis Rheum.

[10] (2018). New insights into CPPD. https://www.the-rheumatologist.org/article/new-insights-into-cppd/.

[11] Rosales-Alexander J, Balsalobre Aznar J, Magro-Checa C (2014). Calcium pyrophosphate crystal deposition disease: diagnosis and treatment. Open Access Rheumatol.

[12] (2018). CPPD arthropathy. https://www.rheumatologyadvisor.com/rheumatology/cppd-arthropathy/article/626324/..

[13] Yavorskyy A, Hernandez-Santana A, McCarthy G, McMahon G (2008). Detection of calcium phosphate crystals in the joint fluid of patients with osteoarthritis - analytical approaches and challenges. Analyst.

[14] Resnick D, Williams G, Weisman M, Slaughter L (1981). Rheumatoid arthritis and pseudo-rheumatoid arthritis in calcium pyrophosphate dihydrate crystal deposition disease. Radiology.

[15] Lomax A, Ferrero A, Cullen N, Goldberg A, Singh D (2014). Destructive pseudo-neuroarthropathy associated with calcium pyrophosphate deposition. Foot Ankle Int.

[16] Akbarnia H (2017). Arthrocentesis, Knee. StatPearls.

[17] Gordon C, Swan A, Dieppe P (1989). Detection of crystals in synovial fluids by light microscopy: sensitivity and reliability. Ann Rheum Dis.

[18] Roane DW, Harris MD, Carpenter MT (1997). Prospective use of intramuscular triamcinolone acetonide in pseudogout. J Rheumatol.

[19] Zhang W, Doherty M, Pascual E (2011). EULAR recommendations for calcium pyrophosphate deposition. Part II: management. Ann Rheum Dis.

[20] Nuki G (2008). Colchicine: its mechanism of action and efficacy in crystal-induced inflammation. Curr Rheumatol Rep.

[21] Nashel DJ (1981). Intravenous administration of colchicine. Arthritis Rheum.

[22] Chollet-Janin A, Finckh A, Dudler J, Guerne PA (2007). Methotrexate as an alternative therapy for chronic calcium pyrophosphate deposition disease: an exploratory analysis. Arthritis Rheum.

[23] Andres M, Sivera F, Pascual E (2012). Methotrexate is an option for patients with refractory calcium pyrophosphate crystal arthritis. J Clin Rheumatol.

[24] Kingsbury S, Tharmanathan P, Adamson Adamson (2013). Hydroxychloroquine effectiveness in reducing symptoms of hand osteoarthritis (HERO): study protocol for a randomized controlled trial. Trials.

[25] Rothschild B, Yakubov LE (1997). Prospective 6-month, double-blind trial of hydroxychloroquine treatment of CPDD. Compr Ther.

[26] Ottaviani S, Brunier L, Sibilia J (2013). Efficacy of anakinra in calcium pyrophosphate crystal-induced arthritis: a report of 16 cases and review of the literature. Joint Bone Spine.

[27] Announ N, Palmer G, Guerne PA, Gabay C (2009). Anakinra is a possible alternative in the treatment and prevention of acute attacks of pseudogout in end-stage renal failure. Joint Bone Spine.

[28] Doherty M, Dieppe P (1983). Double blind, placebo controlled trial of magnesium carbonate in chronic pyrophosphate arthropathy. Ann Rheum Dis.

[29] Aydin M, Arikan M, Togral G, Varis O, Aydin G (2017). Viscosupplementation of the knee: three cases of acute pseudoseptic arthritis with painful and irritating complications and a literature review. Eur J Rheumatol.

[30] AnilKumara AVS, Kumar PG, Shankar S (2009). Role of nuclear medicine in evaluation and management of joint diseases. Indian J Rheumatol.

[31] Knut L (2015). Radiosynovectomy in the therapeutic management of arthritis. World J Nucl Med.

[32] Cheung H, Kurup I, Sallis J, Ryan L (1996). Inhibition of calcium pyrophosphate dihydrate crystal formation in articular cartilage vesicles and cartilage by phosphocitrate. J Biol Chem.

[33] Cheung H, Sallis J, Demadis K, Wierzbicki A (2006). Phosphocitrate blocks calcification-induced articular joint degeneration in a guinea pig model. Arthritis Rheum.

